# Preventing Cardiovascular Disease Among Urban African Americans With a Mobile Health App (the MOYO App): Protocol for a Usability Study

**DOI:** 10.2196/16699

**Published:** 2020-07-09

**Authors:** Herman A Taylor Jr, Sherilyn Francis, Chad Ray Evans, Marques Harvey, Brittney A Newton, Camara P Jones, Tabia Henry Akintobi, Gari Clifford

**Affiliations:** 1 Cardiovascular Research Institute Morehouse School of Medicine Atlanta, GA United States; 2 Nucleus Health Communications Atlanta, GA United States; 3 Emory University Atlanta, GA United States; 4 Georgia Institute of Technology Atlanta, GA United States

**Keywords:** African Americans, mHealth, community-based participatory research, agile design, cardiovascular

## Abstract

**Background:**

Cardiovascular disease (CVD) disparities are a particularly devastating manifestation of health inequity. Despite advancements in prevention and treatment, CVD is still the leading cause of death in the United States. Additionally, research indicates that African American (AA) and other ethnic-minority populations are affected by CVD at earlier ages than white Americans. Given that AAs are the fastest-growing population of smartphone owners and users, mobile health (mHealth) technologies offer the unparalleled potential to prevent or improve self-management of chronic disease among this population.

**Objective:**

To address the unmet need for culturally tailored primordial prevention CVD–focused mHealth interventions, the MOYO app was cocreated with the involvement of young people from this priority community. The overall project aims to develop and evaluate the effectiveness of a novel smartphone app designed to reduce CVD risk factors among urban-AAs, 18-29 years of age.

**Methods:**

The theoretical underpinning will combine the principles of community-based participatory research and the agile software development framework. The primary outcome goals of the study will be to determine the usability, acceptability, and functionality of the MOYO app, and to build a cloud-based data collection infrastructure suitable for digital epidemiology in a disparity population. Changes in health-related parameters over a 24-week period as determined by both passive (eg, physical activity levels, sleep duration, social networking) and active (eg, use of mood measures, surveys, uploading pictures of meals and blood pressure readings) measures will be the secondary outcome. Participants will be recruited from a majority AA “large city” school district, 2 historically black colleges or universities, and 1 urban undergraduate college. Following baseline screening for inclusion (administered in person), participants will receive the beta version of the MOYO app. Participants will be monitored during a 24-week pilot period. Analyses of varying data including social network dynamics, standard metrics of activity, percentage of time away from a given radius of home, circadian rhythm metrics, and proxies for sleep will be performed. Together with external variables (eg, weather, pollution, and socioeconomic indicators such as food access), these metrics will be used to train machine-learning frameworks to regress them on the self-reported quality of life indicators.

**Results:**

This 5-year study (2015-2020) is currently in the implementation phase. We believe that MOYO can build upon findings of classical epidemiology and longitudinal studies like the Jackson Heart Study by adding greater granularity to our knowledge of the exposures and behaviors that affect health and disease, and creating a channel for outreach capable of launching interventions, clinical trials, and enhancements of health literacy.

**Conclusions:**

The results of this pilot will provide valuable information about community cocreation of mHealth programs, efficacious design features, and essential infrastructure for digital epidemiology among young AA adults.

**International Registered Report Identifier (IRRID):**

DERR1-10.2196/16699

## Introduction

### Background

Health inequity is a vast and complex problem, which defies simplistic solutions. Cardiovascular disease (CVD) disparities are a particularly devastating manifestation of health inequity. Despite advancements in prevention and treatment, CVD is still the leading cause of death in the United States [1]. It has a disproportionately worse impact on African Americans, with approximately 65,000 more deaths annually than projections based on age and gender alone would predict [2]. Additionally, research indicates that African American and other US minority populations are affected by CVD earlier than white American adults [3,4]. Many factors contribute to this disparity, including prevalence of CVD risk factors, psychosocial and economic determinants of health (including education, wealth or income, literacy, numeracy neighborhood environment, and others), and insufficient access to quality health and social services [5].

Longitudinal observational studies such as the Framingham Heart Study and the Jackson Heart Study (JHS) have been instrumental in identifying CVD risk factors and tracking outcomes [6]. The JHS in particular has pioneered innovative participant engagement approaches to assess CVD risk factors [7]. This study has focused comprehensively on a marginalized group suffering from health disparities, yielding enhanced understanding of risk and increased precision of risk stratification [8]. The JHS has further emphasized community input and community activism for health and research. The JHS has demonstrated that deep involvement of the target community in building a research initiative can facilitate long-term commitment and retention. A particularly novel and highly successful aspect of the JHS is its training programs [9]. African American high school, college, and graduate students are engaged in subject matter and practicums in epidemiology, bioethics, statistics, and other disciplines essential to the work of the JHS. Graduates of these programs have emerged as a new African American cohort of epidemiologists, physicians, bioethicists, lawyers, and other professionals. They benefited from the affiliation with the JHS and are positioned to impact health disparities across generations.

The lessons of the JHS, learned in the final decade of the 20th century, are timeless and potentially adaptable and scalable in light of rapidly advancing technological developments, which have birthed an age of “digital epidemiology.” Given that mobile phone ownership is near ubiquitous among Americans [10,11], mobile health (mHealth) technologies offer unparalleled potential to prevent or improve self-management of chronic disease. Co-design with the community, an echo of the JHS approach, may hold potential for enhanced cultural congruence, acceptability, and trust of this 21st century approach to population research in marginalized communities. The successful community-driven JHS model suggests an important emergent opportunity to leverage advancements in mobile technology to better identify environmental and behavioral risks as well as design and launch interventions that may be efficacious in reducing risk and improving outcomes. As with the JHS Scholars Program, intentional involvement of young people in the cocreation of this initiative (termed “MOYO,” after the Kiswahili word for heart) will not only introduce them to the potential of connected technology in health but serve to demystify and motivate many of them toward science, technology, economics, and mathematics careers.

### Objectives

MOYO will develop an mHealth approach to eliminating health disparities by (1) co-designing the MOYO app using community-based participatory and agile design methods; (2) integrating electronic health record data generated by traditional methods with data collected “in the wild” (eg, wearables, mobile phone use, local weather, pollution, and geographic location); (3) creating a cloud-based system to allow users to view and track their integrated data over time and improve health care outcomes; and (4) providing educational and community outreach to empower participants to cocreate apps for deployment within the digital ecosystem.

## Methods

### Design

The MOYO study design will be a longitudinal cohort pilot study.

### Theoretical Framework

The theoretical underpinnings of this project combine the health belief model (HBM) and social cognitive theory of mass communication (SCT MC). The HBM and the SCT MC will be used to develop the formative evaluation measures. In addition, the community-based participatory research (CBPR) and the agile software development approaches will be combined to define the user experience and to design the user interface. Multimedia Appendix 1 summarizes the similarities and differences among the principles of these complementary paradigms. These principles provide a framework and guidelines that inform the methodology, development, and design of the MOYO app and the formation of the MOYO app (adapted from the Agile Alliance’s Manifesto [12-14]).

#### Health Belief Model

The HBM will be used to allow for a systematic exploration to understand the target population’s attitudes and beliefs toward the capacity of mobile apps to facilitate behavior change, identify the features of health-focused apps that are the most and least desirable, and define the health-focused app elements that encourage long-term app use. The five relevant constructs of the HBM that will be used are perceived benefits, perceived barriers, perceived threats, self-efficacy, and cues to action. Adhering to CBPR methodology, the Morehouse School of Medicine, the Prevention Research Center, and the Community Coalition Board will work with the MOYO team to develop validated, culturally tailored focus group questions.

#### Social Cognitive Theory of Mass Communication

The SCT MC approaches will augment the use of CBPR and agile design approaches to gain a comprehensive understanding of the design features desired by the target population. The SCT MC, which is based on the original four constructs of the social cognitive theories (ie, self-efficacy, use of incentive motivation, social environment, and reciprocal determinism) will provide a framework for the analysis of psychological mechanisms through which media and communication influences human behavior [15].

#### Community-Based Participatory Research

CBPR is a research approach that emphasizes community-academic partnership and shared leadership in the planning, implementation, evaluation, and dissemination of initiatives [14]. Persons representing our target population will be invited to join the MOYO Project Advisory Board (MPAB). The function of the project advisory board will be to involve community members in all phases of the research process. The MOYO-community partnership will implement the nine principles of CBPR to answer our research questions related to community relevance while addressing community-identified social, structural, physical environmental, and policy priorities. To achieve this measure, the study team will have members of the MPAB define the opportunities and threats to community adoption of the MOYO app. Further, we will use the agile software development framework to address the opportunities and threats presented by the MPAB.

#### Agile Software Development Framework

Consistent with CBPR principles, we did not make a priori assumptions about community needs or preferences in a health-focused app. Therefore, the conventional Waterfall method, typically employed in app design, was rejected in favor of an adaptive software development methodology known as *Agile Design*. A series of “sprints” and “scrums” define the process, with sprints representing the individuals in the process working on focused individual components [16]. Weekly, the research and design teams will assemble (in a scrum) to merge code, refine concepts, and produce an intermediate product for testing. This process will be repeated until a stable minimum viable product is produced. Table 1 compares and contrasts the principles of CBPR and the agile software development framework.

**Table 1:** Key and complementary principles of agile software development and community-based participatory research.

Principles	Community-based participatory research	Agile software development
Promotes collaborative and equitable partnerships in all research phases and involves an empowering and power-sharing process	✓	N/A^a^
Customer (user) satisfaction through early and continuous delivery of useful software	N/A	✓
Recognizes community as a unit of identity	✓	N/A
Builds on strengths and resources within the community	✓	N/A
Welcomes changing requirements, even late in development	N/A	✓
Frequently delivered software (weeks rather than months)	N/A	✓
Facilitates colearning and capacity building among all partners (eg, integrates knowledge and action for mutual benefit of all partners)	✓	✓
Projects are built around motivated individuals who should be trusted to complete the task(s)	N/A	✓
Focuses on problems of relevance to the local community using an ecological approach that attends to multiple determinants of health and disease	✓	N/A
Balances research and action for the mutual benefit of all partners	✓	N/A
Face-to-face conversation is the best form of communication	N/A	✓
Disseminates findings and knowledge gained to the broader community and involves all partners in the dissemination process	✓	N/A
Working software is the primary measure of progress	N/A	✓
Promotes a long-term process and commitment to sustainability	✓	N/A
Sustainable development, able to maintain a constant pace (eg, agile processes promote sustainable development)	N/A	✓
Continuous attention to technical excellence and good design enhances agility	N/A	✓
Simplicity—the art of maximizing the amount of work not done—is essential	N/A	✓
Self-organizing team	N/A	✓
Regular adaptation to changing circumstance	N/A	✓

^a^Not applicable.

### Research Population

African Americans 18-29 years of age who frequent our recruitment locations in Atlanta, Georgia will be invited to participate in the MOYO study.

To be enrolled in the study, potential participants must meet all of the following inclusion criteria: (1) be self-identified as black or African American; (2) be between the ages of 18 and 29 years, inclusively; and (3) own an iOS or Android-based smartphone. Potential study participants will be excluded if they are younger than 18 years.

### Recruitment and Data Collection Procedure

To recruit persons toward the lower end of our age inclusion criteria, we plan to engage high school seniors enrolled in one Atlanta, Georgia-based school district. The school district is characterized as a “large city” school district serving 79.15% self-identified black or African American students [17], and 74.44% of the students receive free or reduced lunch [18]. Additionally, recruitment will take place at the undergraduate serving institutions in Atlanta, Georgia. Each historically black college or university recruitment location is a 4-year institution, where the average cost of attendance is US $34,130 and average estimated salary after attending is US $41,433 [19-21]. In addition, the state college recruitment site is a 4-year institution, where the annual cost of tuition is approximately US $5346, and the estimated salary after attending is US $28,000 [22]. To engage an “out-of-school“ population, participants will be recruited from city of Atlanta job placement and vocational skills training initiatives, which serve Atlanta residents 17-24 years of age.

Furthermore, MOYO will implement community-wide, event-based recruitment strategies. Similar to a *hack-a-thon*, MOYO’s events-based recruitment strategy will convene age-eligible participants to engage in health disparity-based problem solving. At each of these “HealthTech Events,” the participants will be introduced to the impact of national and local disparities in CVD outcomes among urban-African Americans. They will also engage in the design-thinking process to problem solve through the application of technology and analyze key features of mobile- or internet-based development to design, prototype, and implement systems for human-centered computing. The design-thinking process is a problem-solving framework, which was adopted to emphasize human-centered research, diverse teamwork, and rapid prototyping. [23] This user-centered design strategy will result in prototypes of the end user’s version of an optimally designed and maximally functional MOYO app.

### Prospective Data Collection

Prior to entry into the study, participants will be screened for inclusion and exclusion criteria. Once eligibility criteria are met, participants will complete a baseline assessment to be administered via email. The baseline assessment will include questions on demographics (age, gender, self-reported race, education, parents’ education, socioeconomic status); personal and family health history; medication history (prescription and over-the-counter medications); other medical problems, particularly diabetes or uncontrolled hypertension; medical or surgical events and illness history; and history of current mania, psychotic disorder, or substance dependence.

#### Physical Activity

Activity is logged several times a second from the phone’s accelerometer to provide information on sleep and changes in physical activity levels. Notably, we do not collect absolute location to preserve privacy.

#### Mental Health and Well-Being

We will measure the complexity of social network and mood or behavior from phone call logs and text messages. Again, the identity of the sender and recipient, and the content of the conversation or text message is not recorded, only the type of word in a text message as represented in the Linguistic Inquiry and Word Count dictionary [24,25]. Apart from these automatic measures, the user can self-report using standard scales such as the Patient Health Questionnaire-9 [26].

#### Environment

The app also includes data triggered from the user’s location including relative distance travelled from home, weather, pollution levels, local restaurant proximity, and the degree to which the current location is a food desert.

#### Nutrition

Food choices will be logged via text description or photograph.

#### Sleep Quality

Sleep quality metrics will be collected from activity and heart rate data using low-cost wearables.

#### Vital Signs

Blood pressure and heart rate will be collected by typing text or taking a picture of the device such as a health kiosk in a pharmacy. In addition to the app, heart rate and activity can be captured using a wearable.

#### Electronic Health Records

**Figure 1:**
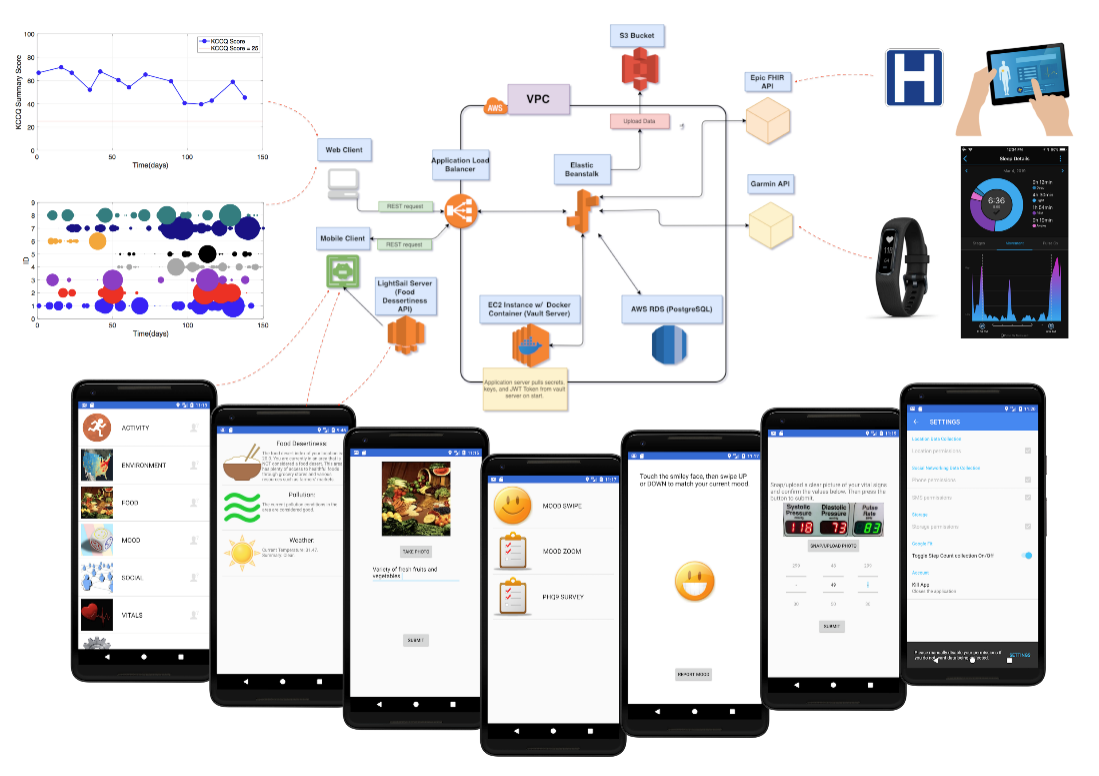
Schematic of app-cloud framework for securely collecting health and activity data. API: application programming interface; AWS: Amazon Web Services; EC2: Amazon Elastic Compute Cloud; FHIR: Fast Healthcare Interoperability Resources; JWT: JSON Web Token; REST: representational state transfer; RDS: Relational Database Service; S3: Amazon Simple Storage Service; SQL: Structured Query Language; VPC: virtual private cloud.

Finally, electronic medical record data can be captured via the Fast Healthcare Interoperability Resource protocol [27]. All data will be pushed to Amazon Web Services in a deidentified manner, and users can log on to see a basic dashboard of their data. At a later date, we intend to engage the users to learn how to manipulate their data via the cloud to build personalized models and improve the dashboard for individuals. Figure 1 illustrates the MOYO smartphone app framework for data collection and secure storage in the cloud.

### Outcome Measures

**Figure 2:**
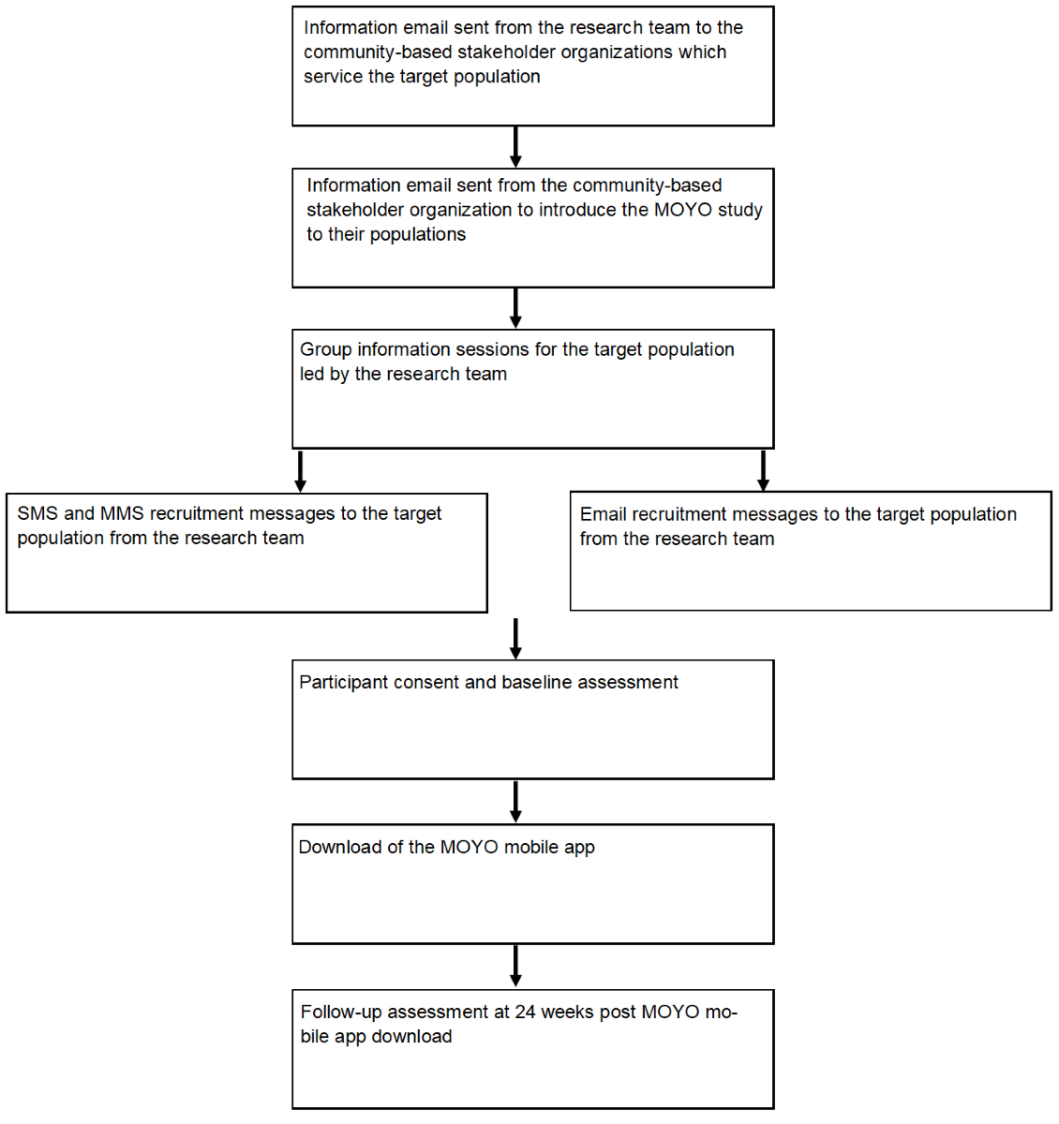
Participant flow and pilot study procedures and assessments. MMS: multimedia messaging service.

The primary outcome measures will assess changes in health-related parameters over a 24-week period as determined by both passive (eg, physical activity levels, sleep duration, social networking) and active (eg, use of mood measures, surveys, uploading of pictures of meals and blood pressure readings) measures. Figure 2 details the participant flow through the MOYO pilot study.

### Statistical Analysis

Data from social network dynamics will standardized metrics of activity, percentage of time away from a given radius of home, circadian rhythm metrics, and proxies for sleep [28]. Together with environment variables (ie, weather, pollution, and socioeconomic indicators such as food quality access), these metrics will be used to train machine-learning frameworks to regress them on the self-reported quality of life indicators. Because this is the first time this has been done in this type of population, it is impossible to estimate effect size and perform a power calculation. In recent works, we showed that this type of approach is powerful in estimating the self-reported quality of life (using a standard scale) in heart failure and patients with bipolar disorder [28,29]. We intend to perform similar analyses on this current cohort to identify changes in mental and physical health as defined by the validated self-report scales.

## Results

This 5-year funded study (2015-2020) is currently in the implementation phase and will run through August 2020. To date, we have enrolled and collected ongoing data from 112 individuals, providing data continuously over a period of up to two years, indicating that there are few barriers to the use of the framework described here. We believe that MOYO can build upon findings of classical epidemiology and longitudinal studies like the JHS by adding greater granularity to our knowledge of the exposures and behaviors that affect health and disease, and creating a channel for outreach capable of launching interventions, clinical trials, and enhancement of health literacy. The study findings will be communicated through peer-reviewed publications and webinars.

## Discussion

We have demonstrated in this phase of our MOYO project that community-based participatory principles can be effectively combined with Agile Design approaches to allow novel mHealth interventions for marginalized communities. This phase of our work is a prologue to the broader deployment and testing of the health app, which aims to better define risk and promote prevention among marginalized groups suffering health inequity.

Health disparities are a persistent, pernicious fact of American life that, in the words of former HHS Secretary Margaret Heckler, are an affront both to our ideals and to the ongoing genius of American medicine [30]. The last 3 decades have seen a growth of literature documenting this problem. Potential solutions to these issues have been far less frequently set forth and realized.

mHealth capabilities and the nearly universal adoption of the cell phone represent a potential “leveling of the (health) playing field.” The collection of critical individual, biometric, and environmental data, at a previously unachievable granularity and scale, combined with uniform and broad access to possible intervention are made possible through technology.

Unlike the often inequitable and spotty American system of health care, cell phone use covers a much wider proportion of the population with increasingly smaller differences in adoption among American ethnoracial groups. Although the digital divide persists, African Americans (and the Latinx population) rely heavily on smartphones for health information and educational material [28]. If leveraged wisely, mHealth could influence bio-psychobehavioral aspects of health and wellness on an unprecedented scale. If applied early in the lifespan, we may disrupt the morbid trajectory, which can take hold in youth and produce excess death and disability by middle age among blacks and other disadvantaged minorities. MOYO is an early effort to establish a platform that will have high rates of use in health disparity communities by adopting the principles that embrace community engagement from inception to implementation. Furthermore, the deep involvement of young users from a historically marginalized group (including training of a selected subset in basic programming) stimulates interest and broadens knowledge in technology for both academic and career opportunities in this underrepresented group.

MOYO leverages four distinct but related research methodologies: CBPR, Agile Design, HBM, and SCT MC. It fuses modern approaches to intervention and technology design to address health inequities and empowers a new generation to improve their health, health literacy, technical literacy, and, as a result, their educational and economic potential. An app designed for deep insights into health requires deep data mining and, therefore, deep trust toward ownership and sustainability among the target population. We adopted a cocreation approach, which involves the young African American population in the technology from the ground-up. By imparting programming skills to a subset of the target demographic, they are becoming advocates and vested creators in the technology. With nonpermissive open-source licensing, it also provides a substrate for the target population to create sustainable and profitable innovations, potentially providing further economic empowerment and technology training opportunities.

## Appendix

Multimedia Appendix 1 Key and complementary principles of agile software development and community-based participatory research. These principles provide a framework and guidelines that inform the methodology, development, and design of the MOYO app and the formation of the MOYO app.

## Abbreviations

AA: African American
CBPR: community-based participatory research
CVD: cardiovascular disease
HBM: health belief model
JHS: Jackson Heart Study
mHealth: mobile health
MPAB: MOYO Project Advisory Board
SCT MC: social cognitive theory of mass communication
