# Supplementary material for: Preventing Cardiovascular Disease Among Urban African Americans With a Mobile Health App (the MOYO App): Protocol for a Usability Study
Source: JMIR Res Protoc. 2020 Jul 9;9(7):e16699. doi: 10.2196/16699 (PMC7380980; doi:10.2196/16699)
Supplement: Multimedia Appendix 1 [file resprot_v9i7e16699_app1.docx]

| **Theoretical Framework** | | |
| --- | --- | --- |
| **Principles** | **Community Based Participatory Research** | **Agile Software Development** |
| **Promotes collaborative and equitable partnerships in all research phases and involves an empowering and power-sharing process** | 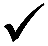 |  |
| **Customer (user) satisfaction through early and continuous delivery of useful software** |  | 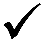 |
| **Recognizes community as a unit of identity** | 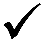 |  |
| **Builds on strengths and resources within the community** | 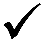 |  |
| **Welcome changing requirements, even late in development** |  | 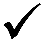 |
| **Frequently Delivered Software (weeks rather than months)** |  | 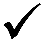 |
| **Facilitates co-learning and capacity building among all partners** **(e.g. integrates knowledge and action for mutual benefit of all partners)** | 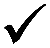 | 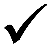 |
| **Projects are built around motivated individuals, who should be trusted to complete the task(s)** |  | 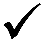 |
| **Focuses on problems of relevance to the local community using an ecological approach that attends to multiple determinants of health and disease** | 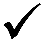 |  |
| **Balances research and action for the mutual benefit of all partners** | 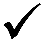 |  |
| **Face-to-face conversation is the best form of communication** |  | 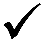 |
| **Disseminates findings and knowledge gained to the broader community and involves all partners in the dissemination process** | 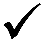 |  |
| **Working software is the primary measure of progress** |  | 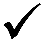 |
| **Promotes a long-term process and commitment to sustainability** | 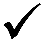 |  |
| **Sustainable development, able to maintain a constant pace (e.g.** **agile processes promote sustainable development)** |  | 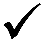 |
| **Continuous attention to technical excellence and good design enhances agility** |  | 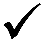 |
| **Simplicity—the art of maximizing the amount of work not done—is essential** |  | 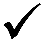 |
| **Self-organizing team** |  | 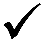 |
| **Regular adaptation to changing circumstance** |  | 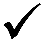 |
